# Psychosocial interventions for depression and anxiety for female adolescents in Sub-Saharan Africa: a protocol for the systematic literature review and lived experience synthesis

**DOI:** 10.1186/s13643-026-03126-9

**Published:** 2026-02-24

**Authors:** Emmanuel Daudi Mollel, Wezi Mhango, Tholene Sodi, Liat Levita, Darya Gaysina

**Affiliations:** 1 School of Psychology, University of Sussex, Sussex, UK; 2 Department of Educational Psychology and Curriculum Studies, University of Dar Es Salaam, Dar Es Salaam, Tanzania; 3 Department of Psychology and Medical Humanities, University of Malawi, Zomba, Malawi; 4 SAMRC-DSI/NRF-UL SARChI Chair in Mental Health and Society, University of Limpopo, Polokwane, South Africa; 5 School of Psychology, University of Sussex, Pevensey 1 Building, Falmer, BN1 9QH UK

**Keywords:** Psychosocial interventions, Depression, Anxiety, Female, Adolescents, Sub-Saharan Africa

## Abstract

**Background:**

Depression and anxiety are common mental health problems among female adolescents worldwide, with higher prevalence rates in Sub-Saharan Africa (SSA). Existing reviews indicate that psychosocial interventions can reduce depression and anxiety in adolescents in SSA. However, no review has been conducted to examine the effectiveness of these interventions for female adolescents specifically.

**Aim(s):**

The primary aim of this study is to systematically review psychosocial interventions for depression and anxiety among female adolescents in SSA.

**Method and analysis:**

A systematic search will be conducted across eight electronic databases—Medline, PubMed, PsycINFO, PsycArticles, Scopus, Cochrane Controlled Register of Trials (CENTRAL), Embase and Web of Science—to identify studies that meet the eligibility criteria. The study selection process will follow the PRISMA guidelines, and will be conducted in Covidence. The quality of the selected studies will be assessed using the Mixed Methods Appraisal Tool. A meta-analysis and narrative synthesis will be applied to the included studies. To complement the findings of the systematic literature review, the lived experience synthesis will be conducted. We will consult the Youth Advisory Group (YAG) composed of up to 10 young females with lived experience of depression and/or anxiety from SSA to get their perspectives, views, and suggestions on the results and dissemination of the prospective systematic review findings.

**Discussion:**

Findings from the review will inform government policies addressing female adolescent mental health problems in Sub-Saharan Africa. It will also inform future research by identifying gaps regarding the effectiveness of psychosocial interventions for female adolescents.

**Conclusion:**

Given the existing gender differences in adolescent mental health, it is imperative to synthesise evidence on the effectiveness of these interventions specifically for female adolescents.

**Systematic review registration:**

PROSPERO CRD420251059110

## Introduction

Adolescence is an extended developmental period characterised by significant brain maturation, as well as substantial physical and social changes [4]. These changes in turn provide adolescents with opportunities for exploration and growth [7]. While this life stage is associated with numerous adaptive features, it is also recognized as a vulnerable period for the development of mental health disorders [57], with most lifetime mental health conditions emerging before the age of 18 [27, 50]. Consequently, adolescent mental health has increasingly become an essential global health and development priority [49, 53]. Globally, it is estimated that 14% of 10–19-year-olds experience mental health disorders, accounting for 15% of the global burden of diseases in this age group [57]. Yet, most of these disorders remain largely unrecognized and untreated [11, 57], thus leading to a high risk of long-term physical and mental impairments in adulthood [4, 7, 53, 57]. Notably, common mental health problems, mainly depression and anxiety, often peak during adolescence [19], and they are among the leading causes of illness and disability among adolescents [55, 57].

Even within this group of adolescents, sex/gender seems to be critical factor in the experience mental health problems. Females than their male counterparts are more likely to report mental health problems [6, 12, 35, 36, 41, 54]. Such that being female is associated with a higher risk of developing common mental health problems particularly depression and anxiety disorders during adolescence [12, 18, 31, 35, 51]. This partly is a result of gender related inequalities such as social isolation, emotional and physical abuse, educational disadvantage, poverty which heightens their risk to developing depression and anxiety [25, 26, 30, 45, 47]. Moreover, hormonal changes associated with puberty disadvantage females more than males. During this period, there is a rapid increase in oestrogen in females and testosterone in males, coinciding with the emergence of sex differences in mental health issues. Prior to puberty, depression occurs at similar rates in males and females, but post-puberty, there is a sharp rise in depressive conditions, with female adolescents being approximately twice as likely to experience depression as males. Early puberty has been identified as a risk factor for depression and anxiety symptoms, particularly in females [4].

Consequently, female adolescents encounter distinct psychological, societal, and developmental challenges [1, 3, 6, 40]. These challenges render them more vulnerable to mental health issues [17, 29, 32, 43], such as depression and anxiety. A recent systematic review shows that the prevalence rate of depression (26.9%) and anxiety (29.8%) among adolescents aged 10–19 years is higher in Sub-Saharan Africa (SSA) [24], compared to the global prevalence rates of these conditions: depression (13%) and anxiety (7–10%). Notably, the prevalence rate of these conditions is significantly higher among female adolescents compared to their male peers in the region [22, 24]. Viewed in its entirety untreated mental health problems lead to social, economic and political disruptions to individuals, their families, communities and nations as untreated mental health problems affect a child’s development, educational attainment and their ability to lead a happy and healthy life [5].

Systematic reviews indicate that psychosocial interventions including cognitive behavioural therapy, interpersonal therapy, psychosocial education group interventions and narrative therapy, effectively reduce depressive and anxiety symptoms among adolescents in SSA [34, 38, 49, 52]. However, these interventions have largely treated adolescents as homogeneous group, overlooking crucial biological sex differences in terms of the prevalence rates, risk factors and the impact of these conditions. Given the existing biological sex differences in adolescent mental health, it is imperative to synthesize evidence on the effectiveness of these interventions specifically for female adolescents.

### The present study

While preparing this protocol, we searched the International Prospective Register of Systematic Reviews, Cochrane Library, the Open Science Framework, and Campbell Collaboration to find out if similar review protocols were registered or completed. Our search found no similar systematic literature reviews that have examined the effectiveness of psychosocial interventions for depression and anxiety specifically for female adolescents in SSA. Thus, our primary aim is to examine the effectiveness of psycho-social interventions in addressing depression and anxiety among female adolescents aged 10–24 in SSA. Evaluating the effectiveness of these interventions is imperative to help researchers recognize and address the specific mental health needs of female adolescents. Our secondary aim is to examine how often RE-AIM components—Reach, Effectiveness, Adoption, Implementation, and Maintenance—are reported in the studies included in this review. While we acknowledge that not all components may have been the focus of the studies that will be included (addressing all is desirable but not always feasible) [13], evaluating their reporting is essential. The RE-AIM framework not only identifies interventions likely to succeed in real-world settings but also highlights those worth sustained investment [15]. Because these components are interdependent in shaping public health impact, incorporating RE-AIM will allow us to synthesize evidence that supports decision-makers in selecting interventions that are feasible, generalizable, and capable of achieving broad public health benefits [16], and allow us to examine factors influencing feasibility, acceptability, and effectiveness.

## Methodology

### Protocol and registration

The protocol was developed in accordance with the Preferred Reporting Items for Systematic Reviews and Meta-Analysis (PRISMA; [33, 39]), which will also guide the review process and the reporting of findings. Further details are provided in Additional file 1. The systematic review protocol was registered with PROSPERO (CRD420251059110) on 27th May 2025.

### Information sources

We will search eight electronic databases: Medline, PubMed, PsycINFO, PsycArticles, Scopus, CENTRAL, Embase, and Web of Science. Our initial search in eight databases identified 6202 papers. Supplementary searches will also be conducted by reviewing the list of references of studies included in the prospective review. We will also contact authors of studies that may be eligible for inclusion but whose full-text articles are not readily accessible. The search will include studies from any setting, length, and providers of the interventions, published in English, peer-reviewed only, and the publication date will not be limited.

### Search strategy

A literature search is a critical element that informs the results of a systematic review and produces the required data for analysis [46]. Thus, the search ought to be robust and reproducible to minimize bias [46]. The keywords for this review will be identified using the PICO framework [21], and their MeSH terms and synonyms will be combined with Boolean operators ("AND" or "OR") to run a search within the mentioned databases. "Speech marks" and truncation* will be used where necessary. The search across the eight databases will be conducted by two authors (EDM & DG), who will integrate the search results and remove all duplicates before the screening is conducted. The pilot search was run from 15 April 2025 to 24 June 2025. The search will be re-run on a 3-month basis for updates. The search across the eight databases will be conducted separately; however, the search results will be merged before the duplicates are removed. The searches for this review will be guided by the statement for reporting literature searches for systematic reviews [46]. Table 1 provides the search strategy that will be used across the eight databases.

**Table 1:** Search strategy across all the databases

Component	Keyword	MeSH term and synonyms
Population	Female, adolescents, Sub-Saharan Africa	MeSH: Female, adolescent Synonyms: female* OR girl* OR "young women" OR adolescen* OR teenager* OR teen* OR youth* OR "young person" OR "young people" OR "young adult" OR student* OR school* OR college* OR university* MeSH: Africa South of Sahara Synonyms: africa* OR "sub-Saharan region" OR "sub-Saharan" OR "sub-Saharan Africa" OR ssa OR "Western Africa" OR "Eastern Africa" OR "Southern Africa" OR "Central Africa"
Intervention	Psychosocial Interventions	MeSH: Psychotherapy Synonyms: "psychosocial interventions" OR "psychological interventions" OR "psycho-social treatment" OR "psycho-social therapy" OR "psycho-social support" OR "cognitive behavioural therapy" OR "CBT" OR "interpersonal psychotherapy" OR "IPT" OR psychoeducation OR "problem-solving therapy" OR "narrative therapy" OR "group therapy" OR "behavioural therapy" OR "self-help intervention" OR "emotional support" OR mindfulness OR counselling
Outcome	Depression and anxiety	MeSH: Depression, Anxiety Synonyms: depress* OR depression OR "depressive disorder" OR dysthymia OR "depressive symptoms" OR "depression symptoms" OR "affective disorder" OR "major depression" OR "major depressive disorder" OR "probable depression" OR anxi* OR anxiety OR "anxiety disorder" OR "anxiety symptoms" OR "generalized anxiety disorder" OR gad OR "panic disorder" OR "social anxiety disorder" OR "emotional problems" OR "internalized problems"

#### Eligibility criteria

The inclusion and exclusion criteria were developed following the PICO framework [21], as summarized in Table 2. The review will include studies that reported the effectiveness of psychosocial interventions for depression and anxiety for female adolescents in SSA.

**Table 2:** Inclusion and exclusion criteria

Component	Include	Exclude
Population	- Studies with female adolescents aged 10–24 - Studies with adolescents whose mean age falls with 24 years - Adolescents and young people from Sub-Saharan Africa	- Studies involved participants under 10 years or older than 24 years - Studies with male participants only - Studies in which the participants' age has not been reported - Adolescents and youth outside Sub-Saharan Africa
Intervention	- Quantitative studies reporting psychosocial interventions for reducing depression and anxiety - Qualitative and mixed-method studies reporting psychosocial interventions for reducing depression and anxiety	- Interventions that are not primarily focused on reducing depression and anxiety symptoms in female adolescents - Non-therapeutic interventions like medication or alternative treatments - Interventions with male and female participants in which female data cannot be independently extracted
Comparison	- Studies from all control arms - Quasi-experimental studies without control groups - Pre and post-intervention studies	- No studies will be excluded based on the characteristics of the control groups
Outcome	- Studies reporting depression and anxiety as primary outcomes - Quantitative studies in which depression and anxiety have been diagnosed or screened using standardised instruments	- Studies not reporting depression and anxiety as primary outcomes - Studies in which depression and anxiety have not been diagnosed or screened using standardised instruments
Study design	- Quantitative studies, qualitative studies, mixed-methods studies, randomised and non-randomised controlled trials, controlled pre and post-interventional studies	- Animal studies, secondary research such as systematic, narrative, book chapters, and meta-analytic reviews
Context	- Peer-reviewed studies conducted in Sub-Saharan, in any settings like schools, hospitals, community health care centres, correctional centres, online learning platforms, homes	- Studies conducted outside Sub-Saharan Africa
Language	- Peer-reviewed studies reported in English	- Peer-reviewed studies not reported in English

### Population and context

The review will include studies with female adolescents aged 10–24 who have been enrolled in an intervention focusing on depression and anxiety. Studies that did not report the age range of participants but reported the mean age that falls within or below 24 years will also be considered for eligibility. The WHO defines adolescent age as being between the ages of 10 and 19. However, the definition of adolescence has been extended to 10–24 years [48]. This expanded and inclusive definition of adolescence is critical for developmentally framing laws, social policies, and services for adolescents [48]. Studies exclusively focused on male adolescents, studies including both males and females without separate data for females, and cohorts outside the 10–24 age range will be excluded from this review. Moreover, the review will include intervention studies from SSA conducted in any setting (schools, hospitals, community health care centres, correctional centres, online learning platforms, homes, etc.). Studies with samples outside SSA will be excluded from this review.

### Intervention(s)

The review will include psychosocial interventions focusing on reducing symptoms of depression and/or anxiety. There will be no restriction regarding the delivery method, type of intervention, length of intervention, intervention setting, and the intervention delivery forms (individual, group, family, peer-led, community-led, professional-led). Furthermore, the review will exclude non-therapeutic interventions like medication, alternative treatments, and interventions with male and female participants where female data cannot be independently extracted from such interventions.

To capture a broad range of interventions, this review will include interventions where the effectiveness of psychosocial interventions has been compared with all types of control arms (no treatment, waitlist, intervention, or any other control condition) and quasi-experimental studies without control groups. Moreover, the review will include interventions where a comparison was conducted between the pre and post-intervention scores.

#### Outcomes

We will include quantitative studies that examined psychosocial interventions where the main outcomes of interest were depression and/or anxiety among female adolescents. The studies will be included if depression and anxiety are assessed through diagnostic status (employing clinical research criteria in line with the DSM or ICD classifications), or if depression and anxiety symptoms are measured using validated self-report instruments and/or screening tools.

We will also consider qualitative studies that examined psychosocial interventions in relation to the prevention of depression and/or anxiety. The review will also examine the reach, adoption, effectiveness, implementation, and maintenance of these interventions. Additionally, from the included qualitative studies, this review will explore why the interventions are considered effective and what makes them effective in reducing depression and anxiety symptoms.

We will extract additional outcomes from studies that reported depression and/or anxiety, including mental health literacy, self-esteem, and social functioning.

#### Study design

The review will include peer-reviewed primary and original research. Specifically, quantitative, qualitative, and mixed-methods studies will be included, such as randomized and non-randomized controlled trials, as well as controlled pre- and post-interventional studies. The review will exclude animal studies, case studies, and secondary research, such as systematic, narrative, and meta-analytic reviews.

#### Data management

All documents related to this systematic review will be securely stored in the shared folder within Drop box of the University of Sussex and will only be accessed by the review team. The feedback from the peer reviewers will be obtained through the journal’s submission system and stored in the shared folder so that all review team members may have access to the folder and responses. We will use Microsoft excel and Microsoft word to manage the review protocol, data extraction forms and the final draft of the manuscript, with the track changes feature enable to ensure that there is transparency in revisions.

#### Study selection

Three independent reviewers will be involved in the study selection process (EDM, WM, and DG). The study selection process will be conducted in two stages, starting with titles and abstracts screening (stage 1) and full-text screening (stage 2) based on the eligibility criteria. We will conduct double-screening on 20% of all records at stages 1 and 2. The purpose of double screening is to assess inter-rater reliability and ensure consistent application of inclusion criteria. The inter-rater reliability will be estimated using Cohen's kappa. Evidence from methodological research indicates that agreement statistics (e.g., Cohen’s κ) and reviewer consistency typically stabilize with samples in the 10–20% range, and that although single screening may miss a small proportion of eligible studies (~ 5%), miss rates are substantially lower when reviewers are experienced (~ 3%) [56]. Major methodological guidelines also endorse partial double screening: the Cochrane Rapid Reviews Methods Group explicitly recommends dual screening of “at least 20%” of abstracts before moving to single-reviewer screening [14], and recent rapid-review guidance similarly supports using a proportion of records (e.g., 20%) for dual screening as a pragmatic and methodologically sound compromise [37]. Therefore, a 20% provides a rigorous and evidence-based balance between quality assurance and resource feasibility. During the double-screening stage, three independent reviewers will meet regularly. Disagreements in the double-screened sample will be resolved by discussion, and screening criteria will be refined where necessary. After achieving acceptable agreement (κ ≥ 0.70), the remaining 80% of records will be screened independently by one reviewer. This approach provides strong quality assurance while optimizing resources.

Covidence software (https://www.covidence.org) will be used to screen titles, abstracts, full-texts/data abstraction, and quality assessment. The study selection process will follow the PRISMA Flow Diagram.

#### Data extraction

As part of the data extraction process, we will conduct 20% parallel data extraction, where at least two independent reviewers will independently extract the same data by following the PICO and RE-AIM frameworks. For the PICO framework, data will be extracted on population characteristics such as geographical location, eligibility criteria, setting, demographics (age, gender, socio-economic status, etc.), sample size, and health status. Intervention characteristics will include design, type of interventions, baseline characteristics, intervention/comparison groups, number of participants allocated to intervention and control groups, frequency of the interventions, modality of the interventions, intervention providers, duration of the intervention, and intervention follow-up. Furthermore, for quantitative and qualitative studies, the main outcomes, such as depression and anxiety, and additional outcomes, such as mental health literacy, self-esteem, and social functioning, will be extracted. The RE-AIM framework will be used to extract data on the reach, effectiveness, adoption, implementation, and maintenance of the interventions. Any disagreements among the reviewers will be resolved by discussing the source(s) of such disagreements, and if a consensus is not reached, a third reviewer will be involved in resolving the arising disagreement. The extracted data will be stored in a spreadsheet (excel or Google Sheets).

#### Quality assessment

The quality of studies will be assessed using the Mixed Methods Appraisal Tool (MMAT) by Hong et al. [23]. Two reviewers (EDM and DG) will independently evaluate the risk of bias in the included studies using MMAT. We chose this tool because our review will include quantitative, qualitative, and mixed method studies, and this tool is designed for systematic reviews that include a variety of study designs [23]. Additionally, the MMAT has been used worldwide as it helps researchers overcome the challenges associated with using different appraisal tools for different designs [10]. Among other things, the tool will determine if the collected data address the research questions, if the findings are adequately derived from the data, if participants adhere to the assigned intervention, if the sample is representative of the targeted population, and if the intervention was implemented as planned. As stressed by this tool, no study will be excluded based on its methodological quality. The overall quality of the included studies will be classified as high, moderate, or low. This will provide a clear understanding of the reliability and confidence that can be placed in systematic review findings.

#### Data synthesis

We will provide a descriptive summary of the study characteristics, such as study design, population characteristics, type of intervention, delivery modality of the interventions, providers of the interventions, outcomes measured, and findings will be presented in figures and tables. For the quantitative studies, a meta-analysis will be conducted to determine the effectiveness of the interventions and factors determining their effectiveness, provided there is sufficient and appropriate data. Inclusion criteria such as randomization, similar population characteristics, and comparable outcomes will be established to ensure the validity and relevance of the included studies.

All statistical analyses will be conducted using meta-analyses packages (i.e., "metan" in stata and "metaphor" in R studio). A random-effects model will be used to account for variability among studies, including intervention types, participant characteristics, intervention providers, and delivery modalities. The standard mean difference will be used to calculate the effect sizes of continuous outcomes of depression and anxiety, and the odds ratio will be used to calculate the effect sizes for the binary or dichotomous outcomes of depression and anxiety. Heterogeneity among studies will be assessed using Cochran's Q test and I^2^ statistics. Forest plot will be used to report the results of a meta-analysis. Publication bias will be assessed using a funnel plot and Eggers regression test. In case publication bias is observed, then Trim and Fill analysis will be employed for adjustment. Sensitivity analysis will be conducted to evaluate the robustness of our review results. The results of the quality assessment will guide the sensitivity analysis where we will be able to understand the impact of quality of studies on the overall results of our review. However, we do not plan exclude studies based on their methodological quality, but rather the extent to which the quality of studies influences the results, and we will report this in our systematic review. Furthermore, we will conduct sub-group analysis by intervention type to see the consistency of their effect sizes on our outcome of interests (depression and/or anxiety).

We also plan to use a narrative synthesis which is an approach to the systematic review and synthesis of findings that uses words and texts to summarize and explain the findings of the synthesis [44]. We acknowledge that narrative synthesis can be problematic because it is associated with the risk that some studies or findings are privileged above others; as such, authors are ought to state clearly in the review protocol how they plan to use narrative synthesis to report the results of primary studies [20]. Thus, in our review, a narrative synthesis will be used to report the types of psycho-social interventions, qualitative findings about interventions effectiveness. Furthermore, it will be used to report quantitative findings on the effectiveness of the interventions in case statistical meta-analysis is not possible due to heterogeneity in the data as a result of diverse designs, outcomes, population and interventions [8, 42]. Narrative synthesis supports the reporting of quantitative data by adopting the textual approach to the process of synthesis to tell the story of the findings from the included studies [8, 44]. Moreover, we will apply narrative synthesis to describe the extent to which the components of the RE-AIM framework were addressed in the included studies.

We will be guided by the narrative synthesis guideline by Popay et al. [44]. This guideline offers advice on conducting narrative synthesis in the context of systematic reviews of research evidence that addresses questions concerning the effects of interventions and those concerned with the implementation of interventions proven effective in experimental settings [44]. The guideline identified the elements that authors can use for a narrative synthesis process of studies included in their review. The elements include developing a theory of how the intervention works, why and for whom, developing a preliminary synthesis of findings of included studies, exploring relationships in the data, and assessing the robustness of the synthesis.

#### RE-AIM components

We will also provide a narrative synthesis on the extent to which studies addressed each of the RE-AIM components, and the synthesis will be based on female data only. The RE-AIM components are reach, effectiveness, adoption, implementation, and maintenance. We will use 34 indicators to assess the reporting of the RE-AIM components. These indicators are based on previous RE-AIM reviews [13, 28] and a review that examined the reporting of RE-AIM components in mental health literacy interventions for female adolescents [2]. A description of how each component will be assessed is provided below.

#### Reach

We will assess reach using five indicators that include participant exclusion criteria (excluded characteristics), percentage of individuals who participate or participation rate, characteristics of participants compared to nonparticipants or target population, qualitative methods to understand reach or recruitment, and methods used to identify the target population.

#### Effectiveness

Effectiveness will be assessed using six indicators. These are measures of primary outcomes with or without comparison (e.g., depression and anxiety), measures of broader outcomes (e.g., mental health literacy, self-esteem, and social functioning), measures of robustness across subgroups (e.g., moderation analysis), measures of short-term attrition rate, intention-to-treat analysis, and use of qualitative methods to understand outcomes.

#### Adoption

Adoption across the studies will be assessed at the setting and staff levels. Setting level adoption will be assessed using four criteria that comprise setting exclusions, percentage of settings approached that participated, characteristics of participating vs. non-participating settings, and use of qualitative methods to understand adoption. At the staff level, adoption will also be assessed using four criteria such as staff exclusions (% or reasons), percentage of staff invited that participated, characteristics of participating vs. non-participating staff or typical staff, and the use of qualitative methods to understand staff participation/staff level adoption.

#### Implementation

We will assess the extent to which the included studies addressed intervention implementation using six indicators. The indicators will include adaptations made to the intervention during the study, extent to which the protocol was delivered as intended, cost of intervention delivery (time or money), consistency in Implementation across staff/time/settings/subgroups, use of qualitative methods to understand implementation, theoretical basis that guided its design and implementation.

#### Maintenance

Maintenance will be assessed at the individual and organization levels. At the individual level, it will be assessed using five indicators. These include measurement of intervention primary outcomes (e.g., depression and anxiety) at 6 months or more after the intervention, measurement of intervention broader outcomes (e.g., mental health literacy, self-esteem, and social functioning) at 6 months or more after the intervention, robustness of data (something about subgroup effects over the long term), long-term attrition rate, and use of qualitative methods to understand long-term impacts. At the organization level, four indicators will be used to assess maintenance including if the intervention is still ongoing at ≥ 6 months post-intervention, if and how the programme was adapted long-term, comments on the sustainability of the intervention, and use of qualitative methods data to understand setting-level institutionalization.

#### Lived experience synthesis

As part of the data synthesis approach, we will consult the Youth Advisory Group (YAG) that will involve up to 10 young females (age: 18–25) with a lived experience of depression and/or anxiety from a SSA country, specifically, Tanzania. The YAG will be asked to share their views on the findings of the systematic literature review; specifically they will assist with the interpretations of results, will help identify gaps in the existing evidence, will suggest directions for future research, and will advise on presenting the research in an accessible and inclusive way, appropriate for the non-academic audience. An advertisement outlining the purpose of the studies, the role of the Youth Advisory Group, and the eligibility criteria will be distributed through the established research networks in Tanzania. Those interested will be asked to contact the first author (EDM) via email or by phone. The researcher's contacts details (email address and phone number) will be included in the information sheet. The youth advisors will be given the information sheet that explains the overall aim of the systematic review. Once they have read and understood, they will be asked if they are willing to take part in the study. If they agree to take part in the study as youth advisors, they will be asked for their verbal consent to record the meetings and use their anonymized commentaries for the prospective review. The consultation meetings with YAG will be conducted in person or virtually via the University of Sussex Zoom account. The zoom link will be sent to participants a day before the meeting and it will have security settings in accordance with the University of Sussex regulations and security procedures. All sessions with YAG will be conducted in focus group format. We plan to conduct up to three consultation meetings with YAG where they will share their views on the preliminary findings of this review. Each session will last between 90 min and 2 h, and will be conducted in either Swahili or English based on the language preferences of the youth advisors.

The focus group discussion sessions are not expected to cause significant psychological distress to the youth advisors. However, if any youth advisor does experience distress during the sessions, the researcher will provide them with a debrief document that contains the name and contacts of relevant organizations and/or support resources where they can reach out for help in case they feel distressed. If youth advisors decide they no longer want to proceed with the study, for any reason, they can withdraw at any time. Youth advisors will be assigned unique codes instead of actual names that will be used to present their session transcripts. Two authors (EDM and DG) will handle the transcription of the sessions, and all transcripts will be stored in an online box folder provided by the University of Sussex. All recordings will be used to produce transcripts and will be deleted upon completion of the systematic review. Ethical approval has been obtained from the Sciences & Technology Cross-Schools Research Ethics Committee (SCITEC) to involve the YAG for lived experience synthesis (ER/EM/727/1).

## Discussion

This review will synthesize and report findings on the effectiveness of psychosocial interventions for depression and anxiety among female adolescents in SSA. Evidence shows that adolescents, particularly females in SSA, are disproportionately affected by common mental health problems [22, 24]. Given the prevalence and the impact of common mental health problems on adolescents, especially females, it is critical that effective interventions are identified and implemented [9]. Notably, various efforts have been implemented in SSA to promote adolescent mental health through the design and implementation of evidence-based mental health interventions. Systematic review evidence shows that these interventions effectively promote positive mental health and prevent common mental health problems among adolescents [34, 38, 49, 52]. However, there is no review targeting the effectiveness of these interventions for female adolescents, as the available reviews on adolescent mental health interventions treated adolescents as a homogeneous group inadequately considering sex differences in this domain [2]. This prospective review will fill this knowledge gap.

Additionally, this will be the first review in the SSA region to report synthesised evidence of these interventions in terms of their reach, adoption, effectiveness, implementation, and maintenance using the RE-AIM framework [15]. This is particularly important as the RE-AIM offers a framework for determining what interventions are worth sustained investment and for identifying those that work in real-world environments [15].

Importantly, the systematic review findings will be presented to the Youth Advisory Group (YAG) with a lived experience of depression and/or anxiety. Through the discussions with the YAG, we will be able to identify the gaps in the existing evidence and suggest the directions for future research. The YAG will help in shaping the prospective review by participating in synthesizing qualitative and quantitative evidence. Their involvement will help to voice their views, which could either confirm or challenge the research team's interpretation of the findings, thereby ensuring that the inferences are relevant to the needs of female adolescents.

The findings from this review will be useful in guiding policymakers in planning and developing policies targeting adolescent mental health. The developed policies should not only address the mental health needs of all adolescents holistically but also cater to the specific mental health needs of each gender. The findings will also lay a foundation for future research to focus on biological sex-specific interventions in addressing common mental health problems among adolescents. We expect relatively limited published research on interventions targeting common mental health problems among female adolescents in SSA might result in having a limited number of eligible papers for consideration in the prospective review. To try to address this and increase the number of studies included in this systematic review, we will contact authors of studies that meet all key inclusion criteria but do not provide separate data for female adolescents. Our aim is to obtain the necessary data to determine whether an analysis specific to female adolescents can be conducted. In addition, we will conduct supplementary searches by reviewing the list of references of studies included in this review. If we find very sparse literature on female-specific intervention in SSA, this will underscore a significant need for investing in sex-specific mental health interventions in SSA.

## Data Availability

Not applicable.
